# Chemical Analysis of Dietary Constituents in *Rosa roxburghii* and *Rosa sterilis* Fruits

**DOI:** 10.3390/molecules21091204

**Published:** 2016-09-09

**Authors:** Meng-Hua Liu, Qi Zhang, Yuan-He Zhang, Xian-Yuan Lu, Wei-Ming Fu, Jing-Yu He

**Affiliations:** 1 Guangdong Provincial Key Laboratory of New Drug Screening, School of Pharmaceutical Sciences, Southern Medical University, Guangzhou 510515, Guangdong, China; menghua_liu@hotmail.com (M.-H.L.); qizhang100@sina.com (Q.Z.); lxy211723@126.com (X.-Y.L.); 2 Bioengineering Research Centre, Guangzhou Institute of Advanced Technology, Chinese Academy of Sciences, Guangzhou 511458, Guangdong, China; zhyuanhe@mail2.sysu.edu.cn

**Keywords:** *Rosa roxburghii*, *Rosa sterilis*, constituents, GC-MS, UFLC/Q-TOF-MS

## Abstract

Both *Rosa roxburghii* and *R. sterilis*, belonging to the Rosaceae, are endemic species in Guizhou Province, China. The fruits of these two species are mixed-used as functional food in the region. Aiming to elucidate the phytochemical characteristics of *R. roxburghii* and *R. sterilis* fruits, the essential oils and constituents in a methanol extract have been analyzed and compared by GC-MS and UFLC/Q-TOF-MS, respectively. As a result, a total of 135 volatile compounds were identified by GC-MS and 91 components were different between *R. roxburghii* and *R. sterilis* fruits; a total of 59 compounds in methanol extracts were identified by UFLC/Q-TOF-MS, including 13 organic acids, 12 flavonoids, 11 triterpenes, nine amino acids, five phenylpropanoid derivatives, four condensed tannins, two stilbenes, two benzaldehyde derivatives and one benzoic acid derivative; and nine characteristic compounds were found between *R. roxburghii* and *R. sterilis* fruits. This systematic study plays an important role for *R. roxburghii* and *R. sterilis* fruits in the product development.

## 1. Introduction

**Figure 1:**
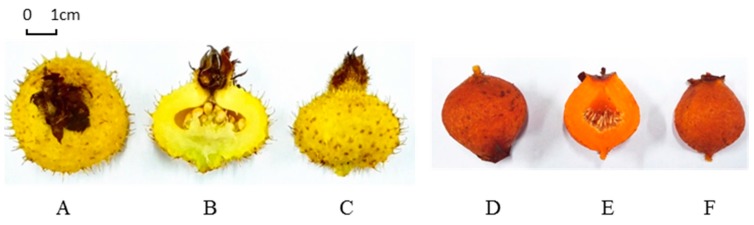
Morphology of *R. roxburghii* (**A**–**C**) and *R. sterilis* (**D**–**F**) fruits.

*Rosa roxburghii* (Figure 1), belonging to the Rosaceae family, originates from the karst areas of Guizhou Province, China and the effect of promoting digestion of its fruit was firstly recorded in “Ben-cao-gang-mu-shi-yi” in 1765 A.D. [1]. Modern pharmacological studies have proven that *R. roxburghii* fruit processes antioxidant, antimutagenic, antiatherogenic and antitumor effects, as well as genoprotective and radioprotective activities [2,3,4,5]. Due to the beneficial effects, a number of phytochemical studies have been performed on this species, and various phytochemicals, including flavonoids, organic acids, triterpenes, amino acids and essential oils have been found in *R. roxburghii* fruit [3,6,7,8,9,10]. Among them, the flavonoids and organic acids in *R. roxburghii* fruit have been widely studied. The total flavonoid content in *R. roxburghii* fruit was 5981–12,895 mg/100 g dry weight, which was approximately 120–360 folds that in citrus [11]. The total content of the six organic acids (malic acid, lactic acid, tartaric acid, citric acid, oxalic acid and succinic acid) in *R. roxburghii* fruit was over 40 mg/g fresh weight [5,12]. Moreover, ascorbic acid, a well-known organic acid, was the most prevalent compound with 4500–6800 mg/100 g dry weight in *R. roxburghii* fruit [13]. To our best knowledge, ascorbic acid is an essential nutrient related to the biosynthesis of collagen and certain hormones, and is a potential substance to reduce the risk of some diseases (e.g., cancer, cardiovascular and neurodegenerative diseases). In view of the high contents and bioactive properties of flavonoids and ascorbic acid, *R. roxburghii* fruit has increasing applications to produce juice, wine and the preserved fruit can be used as a dietary supplement in the health-related industries.

*R. sterilis* (Rosaceae, Figure 1) is a newly found species in Anshun, Guizhou Province, described by Shengde Shi in 1985 [14]. *R. sterilis* have a very close genetic relationship to *R. roxburghii* based on Random Amplified Polymorphic DNA (RAPD) markers [15]. Recently, *R. sterilis* fruit has been mixed with *R. roxburghii* fruit in the food industry due to the fact they come from the same producing region (mainly in Guizhou Province) and similar taste. It is well known that the constituents that are responsible for the flavor and the bioactivities have a great effect on the application of plant materials. Up to now, a few kinds of ingredients in *R. sterilis* fruit, namely essential oils, triterpenes, amino acids and trace elements have been identified [14,16,17,18]. However, it is still difficult to estimate whether *R. sterilis* fruit could be used as a substitute for *R. roxburghii* fruit in the food industry. Thus, it is necessary to elucidate the chemical profiles of *R. roxburghii* and *R. Sterilis* fruits before these two fruits are well developed.

In recent years, gas chromatography-mass spectrometer (GC-MS) and ultra-fast liquid chromatography/quadrupole time-of-flight mass spectrometry (UFLC/Q-TOF-MS) have become powerful technologies for chemical identification in complex extracts due to their high resolution and low detection limit [19,20,21,22]. Therefore, in this study, the essential oils and constituents in methanol extracts of *R. roxburghii* and *R. sterilis* fruits were identified and compared by GC-MS and UFLC/Q-TOF-MS to elucidate their chemical characteristics.

## 2. Results and Discussion

### 2.1. Chemical Analysis and Comparison of Essential Oils by GC-MS

**Figure 2:**
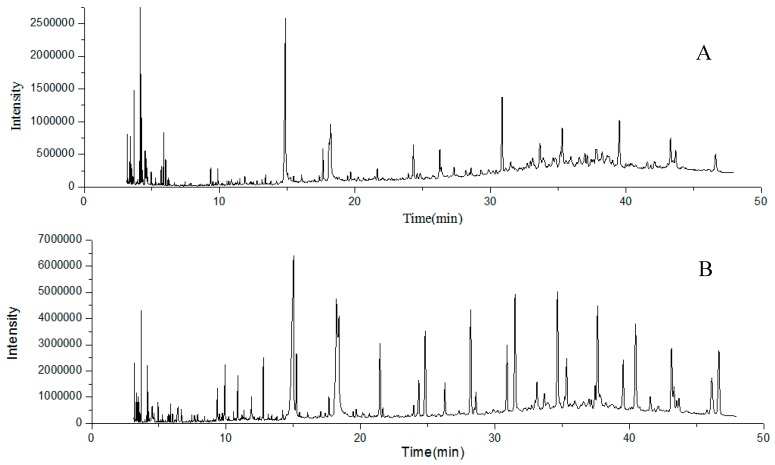
The representative total ion chromatograms of *R. roxburghii* (**A**) and *R. sterilis* (**B**) fruits obtained from GC-MS analysis.

It is well known that essential oils comprise an important part of fruits. In this study, a total of 135 compounds were identified in *R. roxburghii* and *R. sterilis* fruits by GC-MS. As shown in Table 1 and Figure 2, *R. roxburghii* and *R. sterilis* fruits differed in the composition of their essential oils, and only 45 components were shared between them. Interestingly, aliphatic compounds were the major constituents in essential oils of *R. roxburghii* and *R. sterilis* fruits. In *R. roxburghii* fruit, 89 compounds were found, representing 98.88% of the essential oil and the ten components with higher relative peak area were *n*-hexadecanoic acid (16.06%), octadecane (8.16%), 9,12,15-octadecatrien-1-ol (6.66%), nonacosane (6.44%), cholestane (5.24%), β-sitosterol (4.60%), stigmastane (4.44%), tetracosane (3.24%), 9,12-octadecadienoic acid (2.92%), and 2,2′-methylenebis[6-(1,1-dimethylethyl)-4-methyl]phenol (2.78%); 91 compounds represented 93.19% of the essential oil of *R. sterilis* fruit and the ten components with higher relative peak area were *n*-hexadecanoic acid (20.86%), 9,12,15-octadecatrienoic acid (9.06%), octadecane (5.70%), heptadecane (5.38%), nonacosane (5.07%), cholestane (3.93%), stigmastane (3.27%), triacontane (2.88%), 2,2′-methylenebis[6-(1,1-dimethylethyl)-4-methyl]phenol (2.51%) and β-sitosterol (2.36%). The essential oils of *R. roxburghii* and *R. sterilis* fruits have been reported previously [8,11,12,14,15,16]. The essential oil compositions varied highly when different extraction methods were used, such as hydrodistillation, supercritical CO_2_ extraction, solvent extraction and solid-phase microextraction. Liang et al. [23] compared the volatile compounds of *R. roxburghii* fruit extracted by hydrodistillation and solvent extraction, and found that hydrodistillation was the most effective approach for the extraction of long chain fatty acids, such as hexadecanoic acid and 9,12-octadecadienoic acid, which is accord with our results. Zhang et al. [16] separated and identified 41 volatile compounds from *R. roxburghii* fruit by solid-phase microextraction and GC-MS, and confirmed that limonene, ethyl caprylate, ethyl caproate, β-chamigrene and guaiene were the main constituents. A total of 57 volatile compounds from *R. sterilis* fruit have been reported by Jiang et al. [24] and 1,2,3,4-tetrahydro-1,1,6-trimethylnaphthalene, tetradecane, β-selinene, hexanoic acid and dihydro-β-ionol were the main constituents, which were obviously different from those (β-sitosterol, hentriacontane, octacosane, hexanoic acid and 11-(pentan-3-yl) henicosane) obtained by supercritical CO_2_ extraction [25]. Moreover, a higher relative intensity was found in the GC-MS chromatogram of *R. sterilis* fruit, indicating the content of essential oils in *R. sterilis* fruit was more than in *R. roxburghii* fruit using the same extraction method. This was consistent with the reports that extraction rates of essential oils were 1.8% and 0.8 for *R. sterilis* and *R. roxburghii*, respectively [23,25]. Among the 45 shared compounds, most of them have higher relative intensity and peak area in *R. sterilis* fruit than in *R. roxburghii* fruit, except for 5,6-dimethyldecane (**17**). In the present study, more constituents were separated and identified in the essential oils of *R. roxburghii* and *R. sterilis* fruits than in the previous studies using hydrodistillation. Ninety-one volatile compounds were different in these two species, which might explain their different smell and flavor.

Some main constituents in the essential oils have been reported for their pharmacological activities and nutritional values. For example, stigmastane and its derivatives possess anti-herpes virus and anti-inflammatory effects [26]. Meanwhile, 9,12,15-octadecatrienoic acid (linolenic acid), known as a vascular scavenger, has preventative effects against cardiovascular diseases, such as softening heart and brain blood vessels, promoting blood circulation and lowering blood pressure [27,28,29]. Therefore, elucidating the composition of the essential oils of *R. roxburghii* and *R. sterilis* fruits is useful for product development.

### 2.2. Chemical Analysis and Comparison of Multiple Constituents by UFLC/Q-TOF-MS/MS

#### 2.2.1. Identification of Constituents

A total of 59 compounds were identified or tentatively characterized, including 13 organic acids (**1**, **5**–**6**, **8**–**10**, **12**, **16**, **19**, **25**, **28**, **57** and **59**), 12 flavonoids (**18**, **30**–**32**, **34**–**36**, **38**, **44**, **47**, **48** and **49**), 11 triterpenes (**43**, **45**, **46**, **50**–**56** and **58**), nine amino acids (**2**–**4**, **7**, **11**, **13**, **14**, **17** and **20**), five phenylpropanoid derivatives (**26**, **33**, **39**, **41** and **42**), four condensed tannins (**21**, **24**, **27** and **29**), two stilbenes (**37** and **40**), two benzaldehyde derivatives (**15** and **22**) and one benzoic acid derivative (**23**). The detailed information is summarized in Table 2.

Organic acids: Compared with the reference standards, peaks **1**, **5**, **8**, **9**, **10**, **12**, **16**, **19**, **25**, **28**, **57** and **59** were identified directly as lactic acid, malic acid, ascorbic acid, protocatechuic acid, citric acid, *p*-coumaric acid, gallic acid, syringic acid, *p*-hydroxybenzoic acid, caffeic acid, 9,12,15-octadecatrienoic acid and 9,12-octadecadienoic acid, respectively. The characteristic fragments were [M − H − H_2_O]^−^, [M − H − CO_2_]^−^ and [M − H − HCOOH]^−^ in negative ion mode. Compound **6** gave an [M − H]^−^ at *m*/*z* 191.05529, corresponding to its elemental composition of C_7_H_12_O_6_. The high sensitive ions of *m*/*z* 173.0462 resulted from the loss of H_2_O, subsequently the loss of HCOOH yielded the ion *m*/*z* 127.0396. Therefore, compound **6** was tentatively identified as quinic acid [30,31].

Flavonoids: Peaks **30**, **31**, **35**, **44**, **47** and **49** were identified as rutin, isoquercitrin, quercitrin, quercetin, kaempferol and luteolin, respectively. The characteristic fragment of flavonoid glycoside was at *m*/*z* [aglycone]^+^ in positive ion mode and [aglycone]^−^ by the loss of glycoside in negative ion mode. Referring to the compounds reported in *R. roxburghii*, peaks **18** (molecular formula: C_15_H_14_O_7_), **32** (molecular formula: C_27_H_28_O_16_), **36** (molecular formula: C_27_H_28_O_15_) and 38 (molecular formula: C_27_H_28_O_15_) were tentatively identified as epigallocatechin [6], quercetin 3-*O*-[(6-*O*-3-hydroxy-3-methylglutaryl)-β-galactoside] [32], kaempferol 3-*O*-[(X-*O*-3-hydroxy-3-methylglutaryl)-β-galactoside] [32] and kaempferol 3-*O*-[(X-*O*-3-hydroxy-3-methylglutaryl)-β-glucoside] [32], respectively. Peak **34** showed a deprotonated molecule ion at *m*/*z* 433.09253 in negative ion mode. The determination of fragment ion at *m*/*z* 301 confirmed the losses as C_5_H_10_O_4_, suggesting that the existence of xyloside group [9,33]. Peak **48** gave a protonated ion at *m*/*z* 273.07551 and the molecular formula was C_15_H_12_O_5_. According to MS data, fragment ion at *m*/*z* 153 was generated by retro Diels-Alder reaction in the C ring, which is the same as that happened in the standard compounds of quercetin, kaempferol and luteolin. Therefore, it was tentatively identified as dihydroapigenin [33].

Triterpenes: Peak **58** was identified as ursolic acid by comparison with the reference standard. Peaks **43** and **45** were a pair of isomers with a [M − H]^−^ ion at *m*/*z* 665, corresponding to C_36_H_58_O_11_. The fragment ion at *m*/*z* 485, 180 Da less than molecular weight, hinted the presence of a glucose group. Then *m*/*z* 441 appeared behind *m*/*z* 485 in peak **43** of MS spectrum, indicating the presence of a carboxyl group. Considering the retention time and different fragments, the glucose group was not linked to the carboxyl group directly. It was thus tentatively identified as polygalacic acid 3-*O*-β-d-glucopyranoside and peak **45** was tentatively identified as 19α-hydroxyasiatic acid-28-*O*-β-d-glucopyranoside [34]. Peaks **46**, **50** and **52** with *m*/*z* 649.39552 (C_36_H_58_O_10_), 503.33973 (C_30_H_48_O_6_) and 487.34276 (C_30_H_48_O_5_) were tentatively identified as kajiichigoside F1, 1-hydroxyeuscaphic acid, and euscaphic acid, respectively, according to previous reports [7,10,14]. Peaks **51** and **53** had the same molecular formula of C_30_H_46_O_5_. Peak **53** lost neutral ions of H_2_O and CO_2_, then produced fragment ions of *m*/*z* 467 and 441 in MS spectrum, indicating the presence of hydroxyl and carboxyl groups. Therefore, peak **53** was identified as 2α,19α-dihydroxy-3-oxours-12-en-28-oic acid [34,35], and peak **51** was its isomer. Peaks **54** and **55** gave the same molecular formula and fragment ions in their MS spectra, and they were tentatively identified as pomolic acid or an isomer, which needs to be further confirmed by nuclear magnetic resonance (NMR) [7,10,14]. Peak **56** gave a [M − H]^−^ ion at *m*/*z* 469.33187 (C_30_H_46_O_4_). The fragment ions at *m*/*z* 451 and 407 were produced by the loss of H_2_O and the subsequent loss of CO_2_, and it was therefore tentatively identified as 2α,3β-dihydroxylup-20(29)-en-28-oic acid [34].

Amino acids: Nine amino acids identified in the two fruits have been confirmed with reference standards, and they were serine (**2**), arginine (**3**), proline (**4**), valine (**7**), tyrosine (**11**), isoleucine (**13**), leucine (**14**), phenylalanine (**17**) and tryptophan (**20**) [18].

Phenylpropanoid derivatives: Peaks **26** and **41**, a pair of isomers, gave a [M + H]^+^ ion at *m*/*z* 407.16 (C_21_H_26_O_8_) and the fragment ion of *m*/*z* 245, 162 Da lost from the precursor ion, corresponding to a glucose group. Thus, they were identified as *erythro*-guaiacylglycerol β-sinapyl ether and *threo*-guaiacylglycerol β-sinapyl ether, but the configuration of the chiral carbon cannot be confirmed without NMR [26]. Peak **33** gave a [M − H]^−^ ion at *m*/*z* 417.15509, with a molecular formula C_22_H_26_O_8_ by TOF–MS. The fragment ion at *m*/*z* 181 was produced by the dissociation of the furan ring; it therefore was identified as diasyringaresinol [33]. Peak **39** showed a [M − H]^−^ ion at *m*/*z* 435.12995 (C_21_H_24_O_10_). The fragment ion of *m*/*z* 273 unequivocally illustrated the existence of a glucose group. Thus it was tentatively identified as phlorizin [36]. Peak **42** with a protonated [M + H]^+^ ion at *m*/*z* 359.14831 (C_20_H_22_O_6_) was identified as pinoresinol compared with a previous report [36].

Condensed tannins: In the MS spectra, four condensed tannins were identified on the basis of a fragmentation pattern with successive loss of 288 Da corresponding to the loss of catechin units (C_15_H_12_O_6_) [37]. According to previous reports [35,36], they were tentatively identified as procyanidin B1 (**21**), procyanidin B2 (**24**), fisetinidol-(4α,8)-catechin (**27**) and procyanidin B3 (**29**) with the depronated ions at *m*/*z* 305.06686, *m*/*z* 577.13504, *m*/*z* 577.13567, *m*/*z* 561.14044 and *m*/*z* 577.13543, respectively.

Miscellaneous compounds: Peak **15** was exactly identified as vanillin by comparison with a reference standard [33]. Peak **22** exhibited a deprotonated ion at *m*/*z* 181.05076 corresponding to molecular formula of C_9_H_10_O_4_. The fragment ions at *m*/*z* 163, 135 and 119 indicated the existence of hydroxyl, carbonyl and methoxyl groups. It was therefore identified as syringaldehyde [33]. Peak **37** displayed a [M − H]^−^ ion at *m*/*z* 389.12421 (C_20_H_22_O_8_), and the product ion of *m*/*z* 273 hinted the presence of glucose group. It was tentatively identified as piceid [36]. Peak **40** with C_15_H_12_O_2_ gave fragments at *m*/*z* 197 and 185 in its MS spectrum, which were generated by losing CH_2_O and subsequently H_2_O, and was identified as 3-methoxy-5-hydroxy-stilbene [36]. Peak **23** was 162 Da more than peak **25**, and the product ions were very similar to peak **25**. Thus it was identified as 4-hydroxybenzoic acid-4-*O*-β-d-glucopyranoside [36].

#### 2.2.2. Comparison of Multiple Constituents

**Figure 3:**
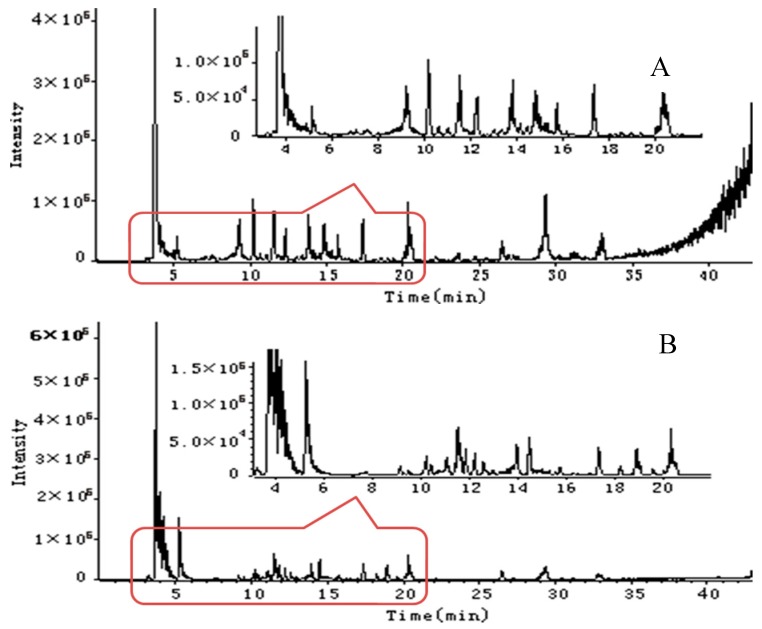
The representative total ion chromatograms of *R. roxburghii* (**A**) and *R. sterilis* (**B**) fruits obtained from UFLC/Q-TOF-MS in negative ion mode.

**Figure 4:**
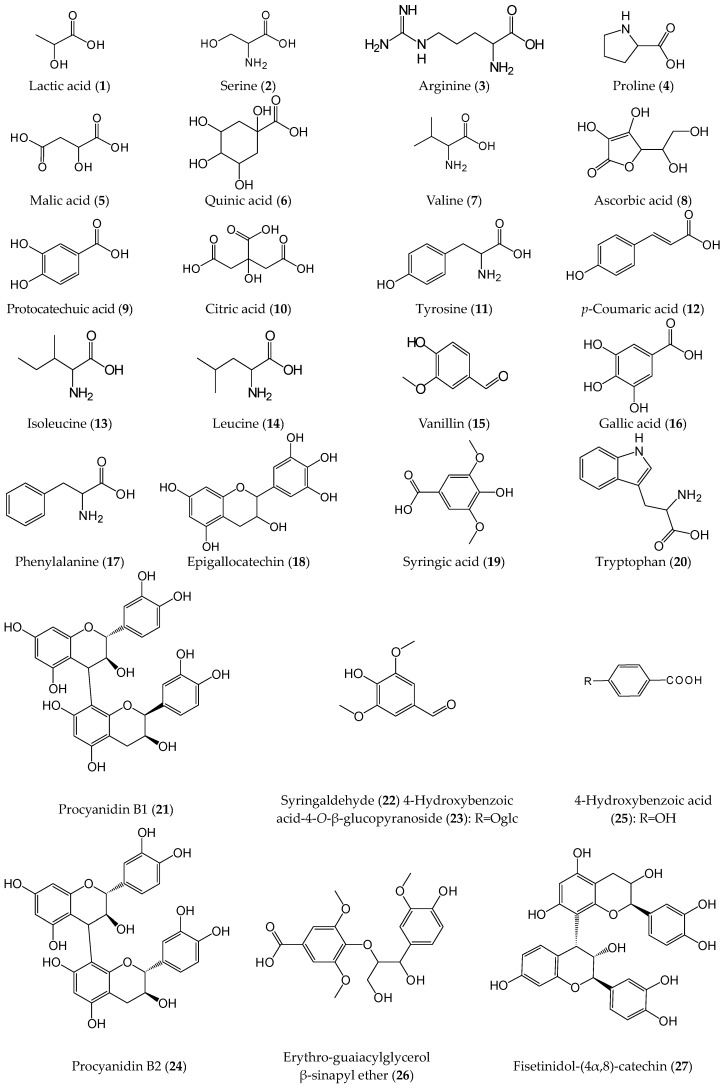

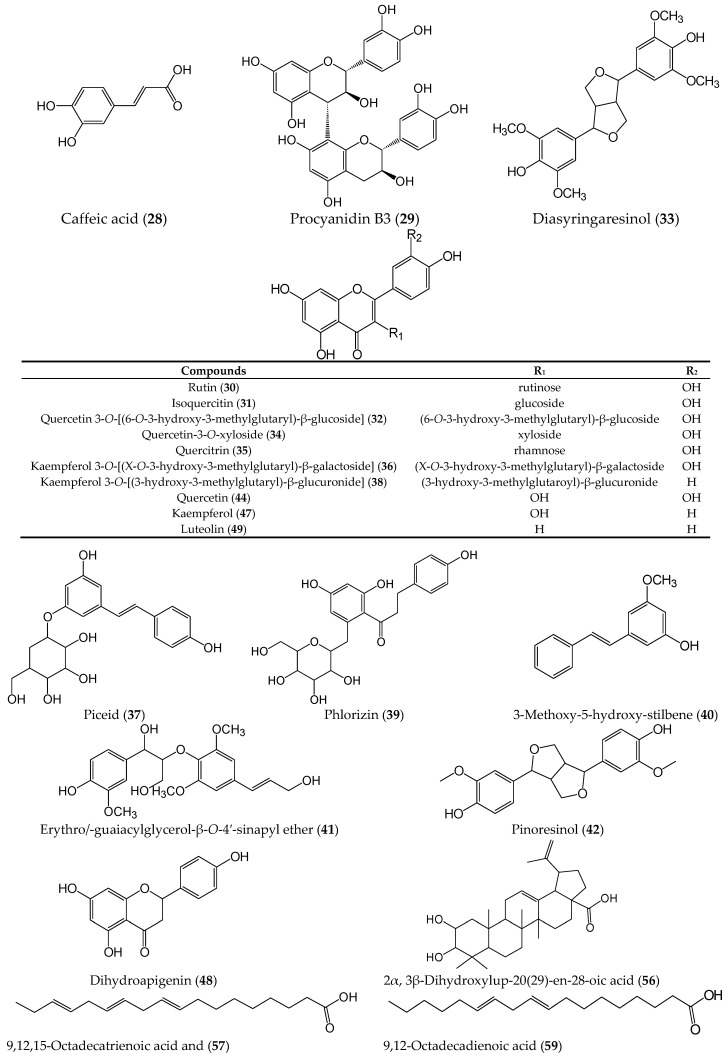

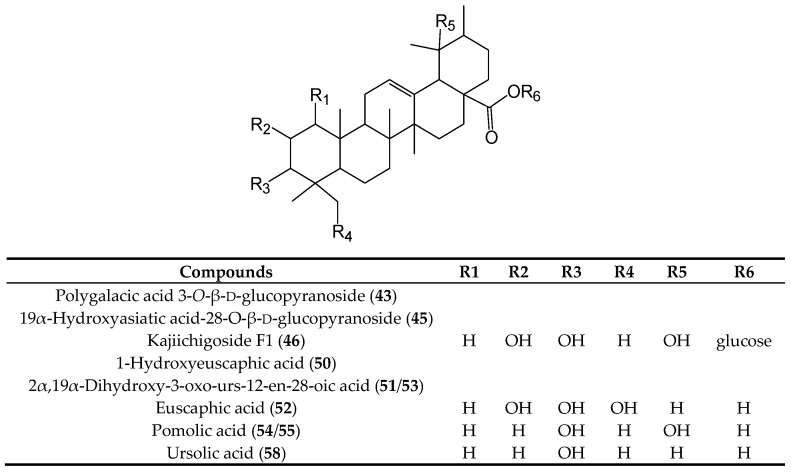
Chemical structures of compounds identified in the methanol extracts of *R. roxburghii* and *R. sterilis* fruits.

UFLC/Q-TOF-MS has become a popular method to analyze constituents in complex systems due to the provided precise molecular weight and fragment characteristic information. Chemical profiles of *R. roxburghii* and *R. sterilis* fruits showed obviously differences based on the representative negative signals (Figure 3). The structures of identified compounds were shown in Figure 4.

Organic acids were the main chemotypes reported in *R. roxburghii* fruit, especially its high ascorbic acid level. Huang et al. demonstrated that the content of ascorbic acid in *R. roxburghii* fruit is higher than that in most common fruit crops, e.g., tomato (~20 mg), strawberry (~50 mg), and kiwifruit (~100 mg) [38]. In this study, a high content of ascorbic acid was also found and determined by a LC-MS method [39]. Comparing the difference of organic acid compositions between *R. roxburghii* and *R. sterilis* fruits, compound **57**, namely 9,12,15-octadecatrienoic acid was only detectable in *R. sterilis* fruit, which was consistent with the result of GC-MS; whereas, syringic acid (**19**) was only found in *R. roxburghii* fruit rather than *in R. sterilis* fruit.

Flavonoids were the second main constituents in both *R. roxburghii* and *R. sterilis* fruits. The radioprotective effects of flavonoids from *R. roxburghii* fruit has been proved by cell model and animal experiments [5]. There were 11 flavonoids, including quercetin, kaempferol and their derivatives found in *R. roxburghii* and *R. sterilis* fruits. However, epigallocatechin (**18**) was only found in *R. sterilis* fruit rather than in *R. roxburghii* fruit.

More than twenty triterpenes have so far been previously found in the Rose family [34]. Triterpenes were other main constituents in *R. roxburghii* fruit with α-glucosidase inhibitory activity [40]. Liang et al. [14] reported that four kinds of triterpenes were isolated from *R. sterilis* fruit three decades ago. In this study, eleven triterpenes were shared between *R. roxburghii* and *R. sterilis* fruits. In addition, polygalacic acid 3-*O*-β-d-glucopyranoside (**43**), 19a-hydroxyasiatic acid-28-*O*-β-d-glucopyranoside (**45**), 2α,19α-dihydroxy-3-oxo-urs-12-en-28-oic acid (**53**), 2α,3β-dihydroxylup-20(29)-en-28-oic acid (**56**) and ursolic acid (**58**) were reported for the first time in *R. sterilis* fruit.

Amino acids as nutrients have various functions in human beings. In addition, the amino acid composition plays an important role affecting the flavor of fruits. In term of the types of compounds, there was no difference between *R. roxburghii* and *R. sterilis* fruits [18].

A total of five phenylpropanoid derivatives were identified in *R. roxburghii* and *R. sterilis* fruits. Among them, diasyringaresinol (**33**) and phlorizin (**39**) were only found in *R. roxburghii* fruit.

Tannins are a kind of secondary metabolite common reported in the genus Rosa, and most of them are characterized by catechin and epicatechin as constitutive units. For example, Yan et al. [36] reported five condensed tannins in *R. laevigata*, namely procyanidin B3, fisetinidol-(4α,8)-catechin, guibourtinidol-(4α,8)-catechin, *ent*-isetinidol-(4α,6)-catechin, fisetinidol-(4β,8)-catechin. In this study, procyanidin B1 (**21**), procyanidin B2 (**24**), and procyanidin B3 (**29**) were commonly found in *R. roxburghii* and *R. sterilis* fruits. Interestingly, fisetinidol-(4α,8)-catechin (**27**) was only found in *R. sterilis* fruit.

Five miscellaneous compounds, including two benzaldehyde derivatives (**15** and **22**), two stilbenes (**37** and **40**) and one benzoic acid derivative (**23**), were detected in this study. Except for the two stilbenes, the presence of the three compounds in *R. roxburghii* and *R. sterilis* fruits was different although they have been found in Rosa species before. Vanillin (**15**) and syringaldehyde (**22**) were only detected in *R. roxburghii* fruit and 4-hydroxybenzoic acid-4-*O*-β-d-glucopyranoside (**23**) only existed in *R. sterilis* fruit.

Generally, 50 phytochemicals existed in both *R. roxburghii* and *R. sterilis* fruits. Five characteristic compounds, including vanillin (**15**), syringic acid (**19**), syringaldehyde (**22**), diasyringaresinol (**33**) and phlorizin (**39**) were found in *R. roxburghii* fruit and the other four compounds, including epigallocatechin (**18**), 4-hydroxybenzoic acid-4-*O*-glucopyranoside (**23**), fisetinidol-(4α,8)-catechin (**27**), and 9,12,15-octadecatrienoic acid (**57**) were only detectable in *R. sterilis* fruit. The nine characteristic phytochemicals were considered to be potential markers for discriminating *R. roxburghii* fruit from *R. sterilis* fruit in quality control. It is general known that phytochemicals are responsible for the bioactivities of medicinal plants. Clarifying the characteristic phytochemicals in *R. roxburghii* and *R. sterilis* fruits is very important for their development and application in health-related industries.

## 3. Materials and Methods

### 3.1. Chemicals and Reagents

Methanol (HPLC grade) and *n*-hexane (GC grade) were purchased from Merck KGaA (Darmstadt, Germany). Distilled water was from Guangzhou Watson’s Food & Drinks Co., Ltd. (Guangzhou, China). The other reagents used were of analytical grade and were used without any further purification. Gallic acid, 4-hydroxybenzoic acid, caffeic acid, protocatechuic acid, isoquercetin, quercitrin, quercetin and kaempferol were purchased from the National Institute for the Control of Pharmaceutical and Biological Products (Beijing, China). Lactic acid, malic acid, ascorbic acid, citric acid, tryptophan, rutin and luteolin were purchased from Aladdin Industrial Corporation (Shanghai, China). Serine, arginine, proline, valine, tyrosine, leucine, isoleucine, phenylalanine, p-coumaric acid, vanillin and syringic acid were purchased from Sigma-Aldrich Chemical Co. (St. Louis, MO, USA). *n*-Hexadecanoic acid, 9,12,15-octadecatrienoic acid, 9,12-octadecadienoic acid, and 9-octadecenoic acid were purchased from Dr. Ehrenstorfer GmbH (Augsburg, Germany). The purities of all compounds were over 95%, as determined by the area normalization method using HPLC or GC.

### 3.2. Plant Material

*R. roxburghii* and *R. sterilis* were cultivated in Guizhou province and their fruits for each species were collected from five cultivars in July (*R. roxburghii*) and October (*R. sterilis*), 2015, respectively. All of the specimens were exactly identified by the authors and stored in −80 °C in Guangzhou Institute of Advanced Technology, Chinese Academy of Sciences, China.

### 3.3. GC-MS Experiment

#### 3.3.1. Sample Preparation

The fresh fruit was washed three times with distilled water and cut randomly into small pieces. Then 100 g of the chipped sample (20 g from each cultivar) was stirred in a bullet blender (Jiuyang Co., Ltd., Shandong, China) for 1 min. After adding 200 mL distilled water, the sample was subjected to hydrodistillation in a steam distillation vessel for 3 h. The oil was extracted by 2 mL hexane and dried with excess anhydrous sodium sulphate. Finally, the oil extract was filtered by 0.22 μm microporous membrane and stored at 4 °C before injection [41].

#### 3.3.2. Instrument Conditions

The analysis was carried out with an 7890A-5975C GC-MSD instrument (Agilent, Santa Clara, CA, USA). Separations were performed using an Agilent HP-5MS capillary column (30 m × 250 mm × 0.25 μm). The GC oven temperature was programmed from 50 °C (1 min isothermal) to 160 °C at 15 °C·min^−1^, and then to 280 °C at 5 °C·min^−1^ (5 min isothermal). A 5 μL sample solution was injected into GC system without split. Helium was the carrier gas with 2 mL/min. The injector temperature was set to 280 °C. The MS was operated in full scan mode (50–500 amu at 0.5 scan/s) with electron ionization mode at 70 eV.

#### 3.3.3. Data Analysis

Data were evaluated by MSD ChemStation E.02.02.1431 software (Agilent). The identification of the compounds was carried out by comparing with data known from the literature, the NIST 05 library (NIST Mass Spectral Database, PC-Version 5.0, 2005, National Institute of Standards and Technology, Gaithersburg, MD, USA) and authentic standards. Percentage data of the total ion current chromatograms were calculated by the area normalization method without applying response factor correction and shown as mean ± standard deviation (SD).

### 3.4. UFLC/Q-TOF-MS Experiments

#### 3.4.1. Sample Preparation

Three hundred gram of fresh fruits (60 g from each cultivar) were washed three times with distilled water and cut into small pieces (thickness about 0.1 cm) and then lyophilized by Modulyo vacuum freeze-drying (Thermo Fisher Scientific Inc., Waltham, MA, USA). During the whole freeze-drying process, the temperature was kept at −50 °C with vacuum degree of 10 mbar for 48 h. Then the dried samples were crushed by Media BM252C blender (Midea Group Ltd., Guangdong, China) and stored at −20 °C until use. One gram of the dried fine powder was accurately weighed and extracted with 25 mL methanol twice in a KQ600DE ultrasonic bath (Kunshan, Jiangsu, China) for 30 min at room temperature. After filtered, the combined extract was dilute with methanol to 50 mL and stored at 4 °C for further study.

#### 3.4.2. Instrument Conditions

The multiple constituents in the methanol extraction of *R. roxburghii* and *R. sterilis* fruits were identified by UFLC/Q-TOF-MS method. Chromatographic analysis was performed on a UFLC XR system (Shimadzu Corporation, Kyoto, Japan) equipped with a LC-20AD-XR binary pump, SIL-20AD-XR autosampler and a CTO-20A column oven. The column was an Agilent Eclipse Plus C18 (2.1 mm × 100 mm, 1.8 μm, Agilent), maintained at 35 °C. The sample was eluted at a flow rate of 0.2 mL/min in a linear gradient mode of A (0.1% formic acid:water) and B (0.1% formic acid:methanol): 0–40 min (5%–100% B), then kept for 3 min at 100% B. The injection volume was 5 μL. Mass spectrometry was performed on the triple TOF™ 5600 (AB SCIEX, Foster City, CA, USA) a hybrid triple quadrupole time-of-flight mass spectrometer equipped with ESI source, and mass range was set at *m*/*z* 100–1200. The experiment parameters were as follows: CUR: 35; GS1: 55; GS2l 55; ISVF: 4500; TEM: 550. Nitrogen was used as nebulizer and auxiliary gas. All the acquisition and analysis of data were controlled by the Peak View Software TM V.1.6 (AB SCIEX).

#### 3.4.3. Data Analysis

Compound identification of methanol extracts of *R. roxburghii* and *R. sterilis* fruits was carried out by comparison of MS and collision-induced dissociation (CID) spectral data of analytes with those of reference standards. When standard substances were unavailable, compounds were tentatively identified by precise molecular weight within an accuracy of 5 ppm or less which can be uniquely associated with a specific molecular formula. At the same time, based on the characteristic pyrolysis fragments obtained by CID spectra, compound structures were assigned by comparison with literature data and standards with similar structures.

## 4. Conclusions

This study analyzed and compared the important dietary constituents in *R. roxburghii* and *R. sterilis* fruits, including essential oils, organic acids, flavonoids, triterpenes, amino acids, phenylpropanoid derivatives, condensed tannins, stilbenes, benzaldehyde derivatives and a benzoic acid derivative. The phytochemical profiles and the chemical differences of these two fruits have been generally illustrated. However, a series of *R. roxburghii* and *R. sterilis* fruits, as the various samples, should be collected from the different producing areas for further study. The contents of main constituents and characteristic compounds, along with their bioactivities, need further in-depth study.

## Figures and Tables

**Table 1:** Volatile compounds identified in *R. roxburghii* and *R. sterilis* fruits by GC-MS (*n* = 3).

No.	Rt (min)	Compounds	Molecular Formula	Molecular Weight	Main Mass Fragments	Area% in RR Fruit	Area% in RS Fruit
1	3.12	3-Furaldehyde	C_5_H_4_O_2_	96	67, 39	-	0.01 ± 0.001
2	3.17	Furfural	C_5_H_4_O_2_	96	67, 39	0.67 ± 0.03	0.57 ± 0.02
3	3.34	3-Hexen-1-ol	C_6_H_12_O	100	83, 67, 41	-	0.53 ± 0.01
4	3.40	4-Methyl-octane	C_9_H_20_	128	112, 85, 71, 69, 43	0.83 ± 0.04	0.17 ± 0.01
5	3.41	Ethyl benzene	C_8_H_10_	106	91, 74, 51, 27	-	0.20 ± 0.02
6	3.48	*o*-Xylene	C_8_H_10_	106	91, 74, 51	0.3 ± 0.003	-
7	3.48	*p*-Xylene	C_8_H_10_	106	91, 74, 51	-	0.28 ± 0.02
8	3.67	Styrene	C_8_H_8_	104	78, 51, 27	1.53 ± 0.05	0.04 ± 0.003
9	4.27	5-Methyl-nonane	C_10_H_22_	142	112, 85, 65, 43	0.16 ± 0.003	-
10	4.94	2-Ethyl-1-hexanol	C_8_H_18_O	130	112, 83, 57, 29	0.23 ± 0.01	-
11	4.99	d-Limonene	C_10_H_16_	158	137, 121, 93, 68, 41	0.22 ± 0.01	0.17 ± 0.01
12	5.02	Benzyl alcohol	C_7_H_8_O	108	79, 51	-	0.02 ± 0.002
13	5.07	1,6-Dimethylhepta-1,3,5-triene	C_9_H_14_	122	107, 91, 65, 41	-	0.03 ± 0.002
14	5.16	3,7-Dimethyl-1,3,6-octatriene	C_10_H_16_	136	121, 93, 77, 57	-	0.05 ± 0.01
15	5.69	4,5-Diethyloctane	C_12_H_26_	170	141, 111, 84, 67, 43	0.63 ± 0.02	0.46 ± 0.02
16	5.94	1,2,4,5-Tetramethylbenzene	C_10_H_14_	134	119, 91, 65	-	0.01 ± 0.001
17	6.01	5,6-Dimethyldecane	C_12_H_26_	170	141, 113, 84, 67, 43	0.73 ± 0.02	0.18 ± 0.02
18	6.17	4-Ethyldecane	C_12_H_26_	170	140, 113, 85, 43	0.18 ± 0.01	-
19	6.24	2,6-Dimethyldecane	C_12_H_26_	170	140, 113, 71, 43	0.29 ± 0.01	-
20	6.68	Dodecane	C_12_H_26_	170	141, 112, 85, 57, 41	0.14 ± 0.01	-
21	7.05	2-Carene	C_10_H_16_	136	121, 93, 77	-	0.03 ± 0.003
22	7.22	2,6,10-Trimethyldodecane,	C_15_H_32_	212	183, 155, 127, 85, 57	-	0.02 ± 0.002
23	7.29	1,2,3,4-Tetrahydro-1,1,6-trimethylnaphthalene	C_13_H_18_	174	159, 131, 105, 71	-	0.07 ± 0.01
24	7.46	3-Methylnonadecane	C_20_H_42_	283	253, 169, 141, 113, 85, 57	0.12 ± 0.01	-
25	7.65	2,3-Dihydro-1,1,5,6-tetramethyl-1*H*-indene	C_6_H_7_BClNO_3_	187	159, 128, 91, 71	-	0.26 ± 0.02
26	7.66	Decane	C_10_H_22_	142	99, 71, 43, 27	0.03 ± 0.004	-
27	8.23	1,1,5-Trimethyl-1, 2-dihydronaphthalene	C_13_H_16_	172	157, 141, 115, 77	-	0.07 ± 0.01
28	8.28	Megastigma-4,6(*Z*),8(*Z*)-triene	C_13_H_20_	176	161, 133, 105, 77	-	0.04 ± 0.004
29	8.47	Dichloroacetic acid, tetradecyl ester	C_16_H_30_Cl_2_O_2_	325	196, 168, 139, 111, 83, 65, 43	0.07 ± 0.006	-
30	8.47	1-Tetradecene	C_14_H_28_	196	168, 140, 111, 83	-	0.04 ± 0.003
31	8.53	Tetradecane	C_14_H_30_	198	169, 141, 113, 85, 57	0.03 ± 0.003	-
32	8.74	Alloaromadendrene	C_15_H_24_	204	189, 161, 119, 91, 69	-	0.06 ± 0.004
33	8.99	3,8-Dimethyldecane	C_12_H_26_	170	141, 113, 85, 57	-	0.08 ± 0.01
34	9.04	9-Methylnonadecane	C_20_H_42_	282	267, 238, 168, 140, 113, 85	-	0.03 ± 0.004
35	9.20	2,6-Bis(1,1-dimethylethyl)-2,5-cyclohexadiene-1,4-dione	C_14_H_20_O_2_	220	177, 135, 91, 67	-	0.07 ± 0.01
36	9.51	2,4-Bis(1,1-dimethylethyl)phenol	C_14_H_22_O	206	191, 163, 115, 91, 57	0.13 ± 0.01	0.13 ± 0.02
37	9.60	1-Butyl-2-propylcyclopentane	C_12_H_24_	168	140, 111, 91, 69, 41	0.03 ± 0.004	-
38	9.65	4-Ethoxybenzoic acid ethyl ester	C_11_H_14_O_3_	194	149, 121, 93, 65	-	0.09 ± 0.01
39	9.87	Dodecanoic acid	C_12_H_24_O_2_	200	157, 129, 101, 73, 43	0.54 ± 0.02	2.26 ± 0.09
40	10.15	1-Tricosanol	C_23_H_48_O	340	322, 294, 154, 125, 97, 69, 43	0.08 ± 0.01	-
41	10.22	Hexadecane	C_16_H_34_	226	169, 141, 113, 85, 57	0.12 ± 0.02	-
42	11.26	2,6,10,14-Tetramethylpentadecane	C_19_H_40_	268	253, 183, 141, 113, 85, 57	-	0.20 ± 0.01
43	11.33	Heptadecane	C_17_H_36_	240	169, 141, 113, 85, 57	0.21 ± 0.01	5.38 ± 0.13
44	11.49	3,7,11-Trimethyl-1,6,10-dodecatrien-3-ol	C_15_H_26_O	222	204, 161, 136, 93, 69	0.24 ± 0.02	-
45	11.72	Hexacosane	C_26_H_54_	366	243, 197, 141, 113, 85, 57	-	0.09 ± 0.004
46	11.83	9-Octylheptadecane	C_25_H_52_	352	239, 197, 169, 141, 113, 85, 57	0.09 ± 0.01	-
47	11.86	Tetradecanoic acid	C_14_H_28_O_2_	228	185, 157, 129, 97, 73	0.44 ± 0.02	0.92 ± 0.02
48	12.08	Benzyl benzoate	C_14_H_12_O_2_	212	167, 105, 77, 51	-	0.27 ± 0.01
49	12.23	6-Tetradecanesulfonic acid butyl ester	C_18_H_38_O_3_S	334	196, 127, 91, 71, 52	-	0.08 ± 0.01
50	12.28	3,3,4-Trimethyl-4-*p*-tolylcyclopentanol	C_15_H_22_O	218	200, 163, 147, 119, 91	0.20 ± 0.01	-
51	12.50	2,6,10,14-Tetramethylhexadecane	C_20_H_42_	282	253, 183, 141, 113, 85	0.18 ± 0.01	-
52	13.06	2-Methylheptadecane	C_18_H_38_	254	239, 211, 141, 113, 85, 57	-	0.11 ± 0.01
53	13.25	Cyclohexadecane	C_16_H_32_	224	125, 55	-	0.16 ± 0.02
54	13.40	1,2-Benzenedicarboxylic acid bis(2-methylpropyl) ester	C_16_H_22_O_4_	278	223, 167, 149, 104, 76, 57	0.44 ± 0.03	0.28 ± 0.01
55	13.49	Cyclopentadecane	C_15_H_30_	210	182, 139, 111, 83, 55	0.07 ± 0.001	-
56	13.63	2-Methylhexacosane	C_27_H_56_	380	365, 337, 169, 141, 113, 85	-	0.10 ± 0.01
57	13.68	Hentriacontane	C_31_H_64_	436	169, 141, 113, 85, 57	-	0.14 ± 0.01
58	13.69	8-Heptadecene	C_17_H_34_	238	210, 140, 111, 83, 55	0.08 ± 0.001	-
59	14.21	2,3-Dimethylnonadecane	C_21_H_44_	297	253, 183, 155, 127, 99, 71	0.19 ± 0.01	-
60	14.53	Palmitoleic acid	C_16_H_30_O_2_	254	236, 192, 137, 111, 83	0.77 ± 0.02	-
61	14.57	Oxacycloheptadecan-2-one	C_16_H_30_O_2_	254	236, 194, 138, 111, 83	0.39 ± 0.01	-
62	14.87	*n*-Hexadecanoic acid ^a^	C_16_H_32_O_2_	256	213, 185, 157, 129, 83, 60	16.06 ± 0.25	20.86 ± 0.31
63	15.64	1-Heptacosanol	C_27_H_56_O	396	378, 181, 153, 125, 97, 57	0.29 ± 0.02	-
64	16.06	3,7,11,15-Tetramethylhexadeca-1,3,6,10,14-pentaene	C_20_H_32_	272	229, 191, 119, 93, 69	0.65 ± 0.01	-
65	16.07	3,7,11-Trimethyl-2,6,10-dodecatrien-1-ol	C_15_H_26_O	222	191, 161, 137, 93, 69	-	0.70 ± 0.01
66	16.12	Trispiro[4.2.4.2.4.2.]heneicosane	C_21_H_44_	296	288, 231, 192, 163, 135, 97	0.29 ± 0.01	-
67	16.47	*N*-[4-bromo-n-butyl]-2-piperidinone	C_9_H_16_BrNO	234	205, 154, 97, 43	-	0.10 ± 0.01
68	16.73	2,2-Dimethyl-5-(3-methyloxiranyl)-cyclohexanone	C_11_H_20_O_2_	196	182, 153, 123, 95, 69, 41	0.11 ± 0.008	-
69	16.74	7-Bromomethylpentadec-7-ene	C_16_H_31_Br	302	223, 153, 125, 97, 69	-	0.15 ± 0.01
70	17.03	1-Nonadecene	C_19_H_38_	266	266, 210, 168, 126, 97	0.31 ± 0.01	0.10 ± 0.01
71	17.04	8-Hexadecene	C_16_H_32_	224	196, 153, 125, 97, 69	-	0.60 ± 0.02
72	17.21	Estra-1,3,5(10)-trien-17β-ol	C_18_H_24_O	256	185, 157, 129, 97, 73	-	0.11 ± 0.01
73	17.23	Nonadecyl pentafluoropropionate	C_22_H_39_F_5_O_2_	430	313, 266, 153, 125, 97, 57	0.03 ± 0.001	-
74	17.36	Heneicosane	C_21_H_44_	296	197, 169, 141, 113, 85	0.63 ± 0.02	0.51 ± 0.02
75	17.64	6-Octen-1-ol, 3,7-dimethyl acetate	C_12_H_22_O_2_	198	156, 123, 103, 81	2.33 ± 0.15	-
76	17.94	Trifluoroacetic acid pentadecyl ester	C_18_H_31_F_3_O_2_	336	306, 255, 182, 140, 111, 83	-	0.42 ± 0.02
77	18.09	9,12-Octadecadienoic acid ^a^	C_18_H_32_O_2_	280	236, 150, 123, 95, 67	2.92 ± 0.09	0.59 ± 0.01
78	18.21	9,12,15-Octadecatrien-1-ol	C_18_H_32_O	264	236, 208, 108, 79	6.66 ± 0.14	-
79	18.40	9,12,15-Octadecatrienoic acid ^a^	C_18_H_30_O_2_	278	222, 163, 135, 108, 79	-	9.06 ± 0.17
80	18.66	4-(4-Ethylcyclohexyl)-1-pentylcyclohexene	C_19_H_34_	262	220, 191, 164, 123, 81	0.42 ± 0.01	-
81	18.66	Methyl 6,9,12-hexadecatrienoate	C_17_H_28_O_2_	264	194, 175, 135, 107, 79	-	0.69 ± 0.02
82	18.80	9-Octadecenoic acid ^a^	C_18_H_34_O_2_	282	264, 222, 165, 137, 111, 83	0.43 ± 0.01	0.14 ± 0.01
83	18.83	1-Eicosene	C_20_H_40_	280	252, 182, 153, 125, 97	0.53 ± 0.02	0.10 ± 0.01
84	18.84	*tert*-Hexadecanethiol	C_16_H_34_S	258	224, 165, 111, 57	-	0.74 ± 0.01
85	18.97	Bacchotricuneatin C	C_20_H_22_O_5_	342	245, 191, 145, 112, 71	-	0.14 ± 0.01
86	19.01	*E*-8-Methyl-7-dodecen-1-ol acetate	C_15_H_28_O_2_	240	197, 165, 126, 97, 69	0.08 ± 0.003	-
87	19.01	3-Methylheptadecane	C_18_H_38_	254	225, 169, 141, 113, 85, 57	-	0.12 ± 0.01
88	19.03	1-Chloro-octadecane	C_18_H_37_Cl	288	175, 147, 113, 85, 57	-	0.50 ± 0.01
89	19.15	2-Dodecen-1-yl(-)succinic anhydride	C_16_H_26_O_3_	266	237, 299, 181, 149, 123, 97, 69, 41	0.07 ± 0.001	0.10 ± 0.01
90	19.21	1-(1,5-Dimethylhexyl)-4-(4-methylpentyl)cyclohexane	C_20_H_40_	280	191, 166, 123, 97, 69	0.05 ± 0.001	0.27 ± 0.01
91	19.68	9-Tricosene	C_23_H_46_	322	294, 167, 139, 111, 83	0.95 ± 0.03	0.89 ± 0.02
92	19.83	13-Tetradecen-1-ol acetate	C_16_H_30_O_2_	254	194, 167, 139, 111, 83	0.13 ± 0.01	-
93	20.55	1-Tricosene	C_23_H_46_	322	196, 169, 139, 111, 83, 57	0.05 ± 0.001	-
94	20.92	1,7,11-Trimethyl-4-(1-methylethyl)cyclotetradecane	C_20_H_40_	280	236, 204, 165, 125, 97	0.10 ± 0.01	0.12 ± 0.01
95	21.03	1-Docosene	C_23_H_46_	308	223, 181, 139, 97, 57	0.33 ± 0.01	0.18 ± 0.01
96	21.34	Eicosyl pentafluoropropionate	C_23_H_41_F_5_O_2_	444	426, 280, 182, 153, 125, 97	0.09 ± 0.005	0.16 ± 0.01
97	21.66	Eicosane	C_20_H_42_	282	197, 169, 141, 113, 85	1.10 ± 0.04	2.28 ± 0.10
98	21.91	Docosane	C_22_H_46_	310	197, 169, 141, 113, 85	0.04 ± 0.003	0.37 ± 0.01
99	22.05	Nonadecane	C_19_H_40_	268	197, 169, 141, 113, 85	0.68 ± 0.01	-
100	22.06	Octatriacontyl trifluoroacetate	C_40_H_77_F_3_O_2_	646	181, 139, 97, 57	-	0.46 ± 0.01
101	22.61	Behenyl chloride	C_22_H_45_Cl	344	189, 169, 141, 113, 85	0.44 ± 0.01	0.05 ± 0.004
102	22.72	11,13-Dimethyl-12-tetradecen-1-ol acetate	C_18_H_34_O_2_	282	267, 208, 151, 123, 95, 69	0.10 ± 0.02	0.69 ± 0.02
103	22.72	15-Isobutyl-(13αH)-isocopalane	C_24_H_44_	332	276, 219, 191, 151, 123, 95	-	0.41 ± 0.01
104	24.21	Cyclotetracosane	C_24_H_48_	336	308, 167, 139, 111, 83	0.41 ± 0.01	0.49 ± 0.01
105	24.32	2,2'-Methylenebis[6-(1,1-dimethylethyl)-4-methyl]phenol	C_23_H_32_O_2_	340	284, 177, 149, 121, 91	2.78 ± 0.09	2.51 ± 0.07
106	24.59	Hexadecyloxirane	C_18_H_36_O	268	250, 211, 166, 138, 111, 82	0.71 ± 0.03	-
107	25.48	Dotriacontyl pentafluoropropionate	C_35_H_65_F_5_O_2_	612	594, 448, 181, 139, 97, 57	0.08 ± 0.002	0.13 ± 0.01
108	26.38	Tetratriacontane	C_34_H_70_	478	253, 225, 197, 169, 141, 113, 85	-	0.39 ± 0.02
109	26.66	12-Pentacosene	C_25_H_50_	350	350, 181, 153, 125, 97, 69	0.20 ± 0.01	-
110	27.32	Bis(2-ethylhexyl)phthalate	C_24_H_38_O_4_	390	279, 180, 149, 104, 57	1.60 ± 0.06	-
111	27.95	Eicosyl trifluoroacetate	C_22_H_41_F_3_O_2_	394	376, 325, 280, 153, 125, 97	0.06 ± 0.002	-
112	28.50	Heptacosyl trifluoroacetate	C_29_H_55_F_3_O_2_	492	474, 423, 378, 181, 139, 97	0.09 ± 0.01	-
113	28.58	Hexacosane	C_26_H_54_	366	169, 141, 113, 85, 57	1.11 ± 0.05	1.20 ± 0.06
114	29.31	2-Dodecen-1-yl(-)succinic anhydride	C_16_H_26_O_3_	266	209, 166, 137, 97, 69	1.65 ± 0.06	0.08 ± 0.002
115	29.53	Tricosane	C_23_H_48_	324	197, 169, 141, 113, 85	0.13 ± 0.01	0.53 ± 0.02
116	29.88	Pentacosane	C_25_H_52_	352	211, 169, 141, 113, 85, 57	-	1.56 ± 0.05
117	29.89	2,6,10,14,18-Pentamethyleicosane	C_25_H_52_	352	253, 183, 141, 113, 85	1.15 ± 0.04	-
118	30.64	2,6,10,14-Tetramethyl-7-(3-methylpent-4-enylidene)pentadecane	C_25_H_48_	348	264, 207, 167, 125, 97	0.56 ± 0.02	-
119	30.67	14-Nonacosane	C_29_H_60_	408	378, 181, 153, 125, 97	0.25 ± 0.01	0.10 ± 0.03
120	30.87	Octadecane	C_18_H_38_	254	169, 141, 113, 85, 57	8.16 ± 0.14	5.70 ± 0.09
121	31.05	1-Hexacosene	C_26_H_52_	364	209 ,181, 153, 125, 97	0.46 ± 0.01	0.54 ± 0.02
122	31.59	1-Bromo-11-iodoundecane	C_11_H_22_BrI	362	281, 233, 177, 135, 97	0.31 ± 0.01	0.27 ± 0.01
123	31.62	1-Methyl-4-(1-methylethyl)-3-[1-methyl-1-(4-methylpentyl)-5-methylheptyl]cyclohexene	C_25_H_48_	348	248, 193, 123, 69	0.24 ± 0.01	-
124	32.46	13-Methyl-*Z*-14-nonacosene	C_30_H_60_	420	405, 209, 181, 153, 125, 97	0.78 ± 0.02	-
125	32.98	(5α,14β)-Cholestane	C_27_H_48_	372	259, 218, 176, 149, 109	2.11 ± 0.03	1.66 ± 0.09
126	33.10	Tetracosane	C_24_H_50_	338	169, 141, 113, 85, 57	3.24 ± 0.05	1.00 ± 0.07
127	33.67	Cholestane	C_27_H_48_	372	262, 217, 149, 109, 81	5.24 ± 0.09	3.93 ± 0.10
128	34.33	(5α,13α)-d-Homoandrostane	C_20_H_34_	274	259, 217, 177, 149, 95, 55	1.25 ± 0.04	1.39 ± 0.06
129	35.31	Nonacosane	C_29_H_60_	408	197, 169, 141, 113, 85	6.44 ± 0.21	5.07 ± 0.19
130	35.94	Stigmastane	C_29_H_52_	400	290, 217, 189, 149, 109	4.44 ± 0.18	3.27 ± 0.14
131	37.45	1-Iodo-octadecane	C_18_H_37_I	380	253, 183, 141, 99, 57	2.16 ± 0.08	0.67 ± 0.03
132	37.46	Triacontane	C_30_H_62_	422	197, 169, 141, 113, 85, 57	-	2.88 ± 0.11
133	37.80	28-Nor-17α(H)-hopane	C_29_H_50_	398	383, 355, 218, 191, 137, 109	2.44 ± 0.09	-
134	43.31	β-Sitosterol	C_29_H_50_O	414	381, 329, 255, 213, 145, 81	4.60 ± 0.17	2.36 ± 0.09
135	43.70	(3β,24*Z*)-Stigmasta-5,24(28)-dien-3-ol	C_29_H_48_O	412	379, 314, 281, 229, 202	-	2.04 ± 0.10

^a^ Compounds were confirmed by the reference standards. RR: *R. roxburghii*; RS: *R. sterilis.*

**Table 2:** Compounds identified in the methanol extracts of *R. roxburghii* and *R. sterilis* fruits.

No.	Rt (min)	Molecular Formula	[M + H]^+^	[M − H]^−^	Major Fragment Ions in Positive Mode	Major Fragment Ions in Negative Mode	Identification	Source
**1**	3.28	C_3_H_6_O_3_		89.02442 (0)		71.0233 [M − H − H_2_O]^−^	Lactic acid ^a^	RR, RS
**2**	3.36	C_3_H_7_NO_3_	106.04983 (–0.4)	104.0534 (+0.1)	87.0324 [M + H − NH_3_]^+^		Serine ^a^	RR, RS
**3**	3.4	C_6_H_14_N_4_O_2_	175.11826 (−4.0)	173.10527 (+5.0)	158.0918 [M + H − NH_3_]^+^, 130.0970 [M + H − NH_3_ − CO]^+^, 116.0724 [M + H − CH_5_N_3_]^+^		Arginine ^a^	RR, RS
**4**	3.82	C_5_H_9_NO_2_	116.07018 (−3.7)	114.05579 (−2.3)	70.0677 [M + H − HCOOH]^+^		Proline ^a^	RR, RS
**5**	3.92	C_4_H_6_O_5_		133.01458 (+2.5)		115.0047 [M − H − H_2_O]^−^	Malic acid ^a^	RR, RS
**6**	3.99	C_7_H_12_O_6_	193.06974 (−4.8)	191.05529 (−4.3)		173.0462 [M − H − H_2_O]^−^, 127.0396 [M − H − H_2_O − HCOOH]^−^, 109.0294 [M − H − 2H_2_O − HCOOH]^−^	Quinic acid	RR, RS
**7**	4	C_5_H_11_NO_2_	118.08617 (−0.7)	116.07236 (+5.7)	72.0828 [M + H − HCOOH]^+^		Valine ^a^	RR, RS
**8**	4.09	C_6_H_8_O_6_	177.03933 (−0.2)	175.02491 (+0.6)	129.0187, 111.0080, 95.0138	115.0043 [M − H − C_2_H_4_O_2_]^−^, 87.0103 [M − H − C_2_H_4_O_2_ − CO]^−^	Ascorbic acid ^a^	RR, RS
**9**	5.18	C_7_H_6_O_4_	155.03362 (−1.7)	153.01985 (+3.4)	137.0253 [M + H − H_2_O]^+^	109.0.93 [M − H − CO_2_]^−^	Protocatechuic acid ^a^	RR, RS
**10**	5.23	C_6_H_8_O_7_	193.03373 (−2.8)	191.02033 (+3.1)		155.0029 [M − H − 2H_2_O]^−^, 111.0079 [M − H − 2H_2_O − CO_2_]^−^	Citric acid ^a^	RR, RS
**11**	5.65	C_9_H_11_NO_3_	182.08028 (−4.9)		147.0444 [M + H − H_2_O − NH_3_]^+^, 136.0761 [M + H − HCOOH]^+^, 119.0493 [M + H − C_2_H_3_NO_2_]^+^, 91.099 2[M + H − C_2_H_3_NO_2_ − H_2_O]^+^		Tyrosine ^a^	RR, RS
**12**	5.65	C_9_H_8_O_3_	165.05427 (−2.1)	163.04076 (+4.2)	119.0467 [M + H − HCOOH]^+^, 91.0563 [M + H − HCOOH − H_2_O]^+^		*p*-Coumaric acid ^a^	RR, RS
**13**	5.73	C_6_H_13_NO_2_	132.10173 (−1.3)	130.08736 (0)	86.0981 [M + H − HCOOH]^+^, 69.0722 [M + H − HCOOH − NH_3_]^+^		Isoleucine ^a^	RR, RS
**14**	6.13	C_6_H_13_NO_2_	132.10198 (+0.6)	130.08852 (+9.0)	86.0982 [M + H − HCOOH]^+^, 69.0720 [M + H − HCOOH − NH_3_]^+^		Leucine ^a^	RR, RS
**15**	6.99	C_8_H_8_O_4_	169.04931 (−1.3)	167.03526 (+1.7)	150.9672 [M+H − H_2_O]^+^, 95.0136	109.0268 [M − H − CO − HCOH]^−^	Vanillin ^a^	RR
**16**	7.72	C_7_H_6_O_5_	171.02825 (−3.2)	169.0144 (+0.9)	139.0017, 111.0063	125.0238 [M − H − CO_2_]^−^	Gallic acid ^a^	RR, RS
**17**	8.09	C_9_H_11_NO_2_	166.08574 (−3.1)	164.07209 (+2.4)	120.0809 [M + H − HCOOH]^+^, 103.0548 [M + H − HCOOH − NH_3_]^+^	147.0454 [M − H − NH_3_]^−^, 120.0444 [M − H − CO_2_]^−^	Phenylalanine ^a^	RR, RS
**18**	9.27	C_15_H_14_O_7_	307.08106 (−0.6)	305.06686 (+0.6)		125.0234 [M − H − C_8_H_8_O_4_]^−^	Epigallocatechin	RS
**19**	10.07	C_9_H_10_O_5_		197.04591 (+1.8)		151.0430 [M − H − HCOOH]^−^, 125.0248 [M − H − CO_2_ − H_2_O]^−^	Syringic acid ^a^	RR
**20**	10.14	C_11_H_12_N_2_O_2_	205.09718 (+0.1)	203.08256 (−0.2)	188.0691 [M + H − NH_3_]^+^, 170.0595 [M + H − NH_3_ − H_2_O]^+^, 146.0593, 118.0648	116.0507 [M − H − C_3_H_7_NO_2_]^−^	Tryptophan ^a^	RR, RS
**21**	10.40	C_30_H_26_O_12_	579.14939 (−0.5)	577.13504 (−0.2)	427.1022 [M + H − C_8_H_8_O_3_]^+^, 409.0898 [M + H − C_8_H_8_O_3_ − H_2_O]^+^, 287.0554 [M + H − C_15_H_16_O_6_]+	451.1050, 425.0893 [M − H − C_8_H_8_O_3_]^−^, 407.0783 [M − H − C_8_H_8_O_3_ − H_2_O]^−^, 289.0729 [M − H − C_15_H_12_O_6_]^−^	Procyanidin B1	RR, RS
**22**	10.54	C_9_H_10_O_4_	183.06502 (−0.9)	181.05076 (+0.7)		163.0367 [M − H − H_2_O]^−^, 135.0449 [M − H − H_2_O − CO]^−^, 119.0495 [M − H − CH_2_O − CH_4_O]^−^	Syringaldehyde	RR
**23**	10.72	C_13_H_16_O_8_		299.0775 (+0.9)		137.0241 [M − H − glc]^−^, 93.0358 [M − H − glc − CO_2_]^−^	4-Hydroxybenzoic acid-4-*O*-glucopyranoside	RS
**24**	11.06	C_30_H_26_O_12_	579.14973 (0)	577.13567 (+0.9)	453.1169, 427.1037, 409.0923, 301.0721, 287.0554	289.0730 [M − H − C_15_H_12_O_6_]^−^	Procyanidin B2	RR, RS
**25**	11.73	C_7_H_6_O_3_	139.03867 (−2.2)	137.02506 (+4.7)	111.0440 [M + H − H_2_O]^+^, 95.0135 [M + H − CO_2_]^+^	93.0351 [M − H − CO_2_]^−^	4-Hydroxybenzoic acid ^a^	RR, RS
**26**	12.39	C_21_H_26_O_8_	407.16816 (−4.6)		245.0449[M + H − glc]^+^		Erythro-guaiacylglycerol β-sinapyl ether or threo-guaiacylglycerol β-sinapyl ether	RR, RS
**27**	12.41	C_30_H_26_O_11_		561.14044 (+0.4)		407.0756 [M − H − C_8_H_10_O_3_]^−^, 289.0718 [M − H − C_15_H_12_O_5_]^−^, 273.07006 [M − H − C_15_H_12_O_6_]^−^	Fisetinidol-(4α,8)-catechin	RS
**28**	13.01	C_9_H_8_O_4_		179.03508 (+0.5)		135.0446 [M − H − CO_2_]^−^	Caffeic acid ^a^	RR, RS
**29**	13.29	C_30_H_26_O_12_	579.14959 (−0.2)	577.13543 (+0.5)	439.1030, 427.1038 [M + H − C_8_H_8_O_3_]^+^, 409.0909 [M + H − C_8_H_8_O_3_ − H_2_O]^+^, 301.0738, 287.0554, 271.0620	451.1072, 425.0879 [M + H − C_8_H_8_O_3_]^−^, 407.0789 [M + H − C_8_H_8_O_3_ − H_2_O]^−^, 289.0716 [M − H − C_15_H_12_O_6_]^−^	Procyanidin B3	RR, RS
**30**	14.06	C_27_H_30_O_16_	611.16016 (−0.8)	609.14646 (+0.6)	303.0460 [M + H − rutinose]^+^	301.0333 [M − H − rui]^−^, 271.0238 [M − H − rui − CH_2_O]^−^	Rutin ^a^	RR, RS
**31**	14.72	C_21_H_20_O_12_	465.10249 (−0.6)	463.08802 (−0.4)	303.0504 [M + H − glc]^+^	301.0347 [M − H − glc]^−^, 271.0260, 255.0292, 151.0027	Isoquercitrin ^a^	RR, RS
**32**	15.18	C_27_H_28_O_16_	609.14377 (−2.0)	607.13045 (0)	303.0491	463.0839, 301.0352	Quercetin 3-*O*-[(X-*O*-3-hydroxy-3-methylglutaryl)-β-glucoside]	RR, RS
**33**	15.23	C_22_H_26_O_8_		417.15509 (−1.0)		181.0482 [M − H − C_13_H_16_O_4_]^−^,	Diasyringaresinol	RR
**34**	15.57	C_20_H_18_O_11_	435.09253 (+0.8)	433.07753 (−0.2)	303.0508 [M + H − C_5_H_10_O_4_]^+^	301.0358 [M − H − C_5_H_10_O_4_]^−^	Quercetin-3-*O*-d-xyloside	RR, RS
**35**	15.77	C_21_H_20_O_11_	449.10749 (−0.8)	447.09296 (−0.7)	303.0846 [M + H − C_6_H_12_O_4_]^+^, 151.0380, 123.0429	300.9984 [M − H − C_6_H_12_O_4_]^−^, 285.0406	Quercitrin ^a^	RR, RS
**36**	15.94	C_27_H_28_O_15_	593.14886 (−2.1)	591.13566 (+0.2)	287.0543	529.1312, 489.1046, 447.0920, 285.0309	Kaempferol 3-*O*-[(X-*O*-3-hydroxy-3-methylglutaryl)-β-galactoside]	RR, RS
**37**	16.28	C_20_H_22_O_8_		389.12421 (0)		227.0712 [M − H − glc]^−^	Piceid	RR, RS
**38**	16.36	C_27_H_28_O_15_	593.14843 (−2.8)	591.13617 (+1.1)	287.0544	529.1236, 489.1044, 447.0936, 285.0407	Kaempferol 3-*O*-[(X-*O*-3-hydroxy-3-methylglutaryl)-β-glucoside]	RR, RS
**39**	16.83	C_21_H_24_O_10_		435.12995 (0.6)		273.0781[M − H − glc]^−^, 167.0349	Phloridzin	RR
**40**	17.21	C_15_H_14_O_2_	227.10655 (−0.4)		197.0650 [M + H − CH_2_O]^+^, 185.0997 [M + H − CH_2_O − H_2_O]^+^		3-Methoxy-5-hydroxy-stilbene	RR, RS
**41**	17.35	C_21_H_26_O_8_	407.16945 (−1.5)		389.1582 [M + H − H_2_O]^+^, 371.1472 [M + H − 2H_2_O]^+^, 245.1159 [M + H − glc]^+^, 215.1062, 199.1114		Erythro-guaiacylglycerol β-sinapyl ether or threo-guaiacylglycerol β-sinapyl ether	RR, RS
**42**	18.53	C_20_H_22_O_6_	359.14831 (−1.7)	357.13435 (0)	327.1216 [M − H − CH_4_O]^−^, 313.1057 [M − H − H_2_O − CO]^−^, 253.0856		Pinoresinol	RR, RS
**43**	18.68	C_36_H_58_O_11_		665.39109 (+0.7)		619.3935 [M − H − 2H_2_O]^−^, 485.3284 [M − H − glc − H_2_O]^−^, 441.3399 [M − H − glc − H_2_O − CO_2_]^−^, 357.2748	Polygalacic acid 3-*O*-β-d-glucopyranoside	RR, RS
**44**	18.9	C_15_H_10_O_7_	303.0497 (−0.7)	301.03548 (+0.4)	257.0431, 229.0497, 201.0540, 165.0182, 153.0181, 137.0230	178.9987, 151.0038, 121.0290	Quercetin ^a^	RR, RS
**45**	19.38	C_36_H_58_O_11_		665.39114 (+0.8)		485.3272 [M − H − glc − H_2_O]^−^, 467.3211 [M − H − glc − 2H_2_O]^−^, 351.2653	19α -hydroxyasiatic acid-28-*O*-β-d-glucopyranoside	RR, RSS
**46**	20.39	C_36_H_58_O_10_		649.39552 (−0.3)		487.3427 [M − H − glc]^−^, 469.3319 [M − H − glc − H_2_O]^−^	Kajiichigoside F1	RR, RS
**47**	20.64	C_15_H_10_O_6_	287.05474 (−1.0)		165.0133, 153.0202		Kaempferol ^a^	RR, RSS
**48**	22.10	C_15_H_12_O_5_	273.07551 (−0.9)		153.0181	151.003	Dihydroapigenin	RR, RS
**49**	22.95	C_15_H_10_O_6_	287.05465 (−1.3)	285.04066 (+0.7)	268.9789, 153.0188, 121.0251	229.0563, 187.0393, 169.0649, 143.0520	Luteolin ^a^	RR, RS
**50**	26.48	C30H48O6		503.33793 (+0.2)		485.3320 [M − H − H_2_O]−, 439.3229 [M − H − HCOOH − H_2_O]−, 421.3141 [M − H − HCOOH − 2H_2_O]− 225.1623	1-Hydroxyeuscaphic acid	RR, RS
**51**	27.25	C30H46O5	487.34155 (−0.5)	485.32722 (−0.1)	451.3208 [M + H − 2H_2_O]+, 405.3170 [M + H − 2H_2_O − HCOOH]+, 223.1604, 199.1448, 187.1454	467.3176, 425.23124, 375.3047, 321.2564, 257.2382	2α,19α-Dihydroxy-3-oxo-urs-12-en-28-oic acid isomer	RR, RS
**52**	29.27	C30H48O5	489.35729 (−0.3)	487.34276 (−0.3)	407.3131 [M + H − 2H_2_O − HCOOH]+, 207.1733, 201.1624	469.3302 [M − H − H_2_O]− 443.3533 [M − H − CO_2_]−, 427.3205 [M − H − H_2_O − CO_2_]−, 371.2911	Euscaphic acid	RR, RSS
**53**	33.05	C30H46O5	487.34155 (−0.5)	485.32728 (+0.1)	451.3208 [M + H − 2H_2_O]+, 405.3170 [M + H − 2H_2_O − HCOOH]+, 223.1604, 199.1448, 187.1454	467.3186 [M − H − H_2_O]−, 441.3415 [M − H − CO_2_]−, 423.3280 [M − H − H_2_O − CO_2_]−, 393.3161	2α,19α-Dihydroxy-3-oxo-urs-12-en-28-oic acid	RR, RS
**54**	34.87	C30H48O4		471.34821 (+0.5)		453.3398 [M − H − H_2_O]− 423.3270, 407.3350, 377.2865	Pomolic acid or isomer	RR, RS
**55**	35.53	C30H48O4		471.348 (0)		453.3350 [M − H − H_2_O]−, 407.3315, 377.2888	Pomolic acid or isomer	RR, RS
**56**	35.56	C30H46O4		469.33187 (−1.0)		451.3237 [M − H − H_2_O]−, 407.3304 [M − H − H_2_O − CO_2_]−, 377.3222	2α,3β-Dihydroxylup-20(29)-en-28-oic acid	RR, RS
**57**	40.72	C18H30O2	279.2318 (−0.2)				9,12,15-Octadecatrienoic acid ^a^	RS
**58**	42.58	C30H48O3	457.36755 (−0.2)	455.35307 (−0.6)		407.3335 [M − H − HCOOH]−	Ursolic acid ^a^	RR, RS
**59**	42.68	C18H32O2	281.24735 (−0.6)	279.23322 (+1.0)		261.2124 [M − H − H_2_O]−	9,12-Octadecadienoic acid ^a^	RR, RS

^a^ Compound was confirmed by reference standard. RR: *R. roxburghii*; RS: *R. sterilis.*
